# The role of triage in the prevention and control of COVID-19

**DOI:** 10.1017/ice.2020.185

**Published:** 2020-05-04

**Authors:** Qiaoxia Wang, Xiaoping Wang, Huanping Lin

**Affiliations:** 1 Department of Infectious Disease, Xi’an Central Hospital, Xi’an, China; 2 Department of Medicine, Xizang Minzu University, Xianyang, China; 3 Department of Medicine, Shaanxi University of Chinese Medicine, Xianyang, China

## Abstract

**Objective:**

To prevent and control public health emergencies, we set up a prescreening and triage workflow and analyzed the effects on coronavirus disease 2019 (COVID-19).

**Methods:**

In accordance with the requirements of the level 1 emergency response of public health emergencies in Shaanxi Province, China, a triage process for COVID-19 was established to guide patients through a 4-level triage process during their hospital visits. The diagnosis of COVID-19 was based on positive COVID-19 nucleic acid testing according to the unified triage standards of the *Guidelines for the Diagnosis and Treatment of Novel Coronavirus Pneumonia (Trial version 4)*,^4^ issued by the National Health Commission of the People’s Republic of China.

**Results:**

The screened rate of suspected COVID-19 was 1.63% (4 of 246) in the general fever outpatient clinic and 8.28% (13 of 157) in the COVID-19 outpatient clinic, and they showed a significant difference *(P* = .00).

**Conclusions:**

The triage procedure effectively screened the patients and identified the high-risk population.

Since January 2020, many cases of viral pneumonia have emerged in Wuhan City, Hubei Province, China, and the disease has rapidly spread to many provinces and cities across China.^1^ On January 23, 2020, 3 patients were confirmed to have newly imported coronavirus disease 2019 (COVID-19) in Shaanxi Province.^2^ On January 25, 2020, Shaanxi Province launched the level 1 emergency response for public health emergencies.^3^ Xi’an Central Hospital also adopted a series of measures to strengthen the prescreening and triage of COVID-19–induced pneumonia and launched quarantine wards on Tang Fang Street on February 1, 2020.

## Methods

### Triage epidemiological criteria

According to the guidelines, the uniform epidemiological triage criteria included the following individuals: (1) those with a history of visiting or living in other provinces, cities, or areas, where local cases continued to spread within 14 days before the disease onset; (2) those who had had contact with patients with fever or respiratory symptoms from other provinces, cities, or areas where local cases continued to spread within 14 days before the disease onset; and (3) those with clustered onset or were epidemiologically linked to COVID-19. The COVID-19 outbreak originated in the Wuhan area. In this study, patients with a history of visiting or living in Wuhan or exposure to the Wuhan population were considered the high exposure-risk group and other patients were considered the low exposure-risk group.

### Clinical prescreening and triage criteria

According to the guidelines, the clinical prescreening and triage criteria included the following individuals: (1) those with fever/respiratory symptoms; (2) those with multiple patched shadows and interstitial changes located in the lung periphery on chest computed tomography (CT) scan; and (3) those with normal or reduced total white blood cell count or reduced lymphocyte count in the early stage of disease onset. Before being qualified for a prescreening or triage position, all medical and nursing staff personnel received the systematic and strict training. COVID-19–positive cases were confirmed based on a positive SARS-CoV-2 nucleic acid test. Suspected cases were excluded after nucleic acid tests for SARS-CoV-2 were negative for 2 consecutive tests, with a sampling interval of at least 1 day.

### Triage system

**Fig. 1.**
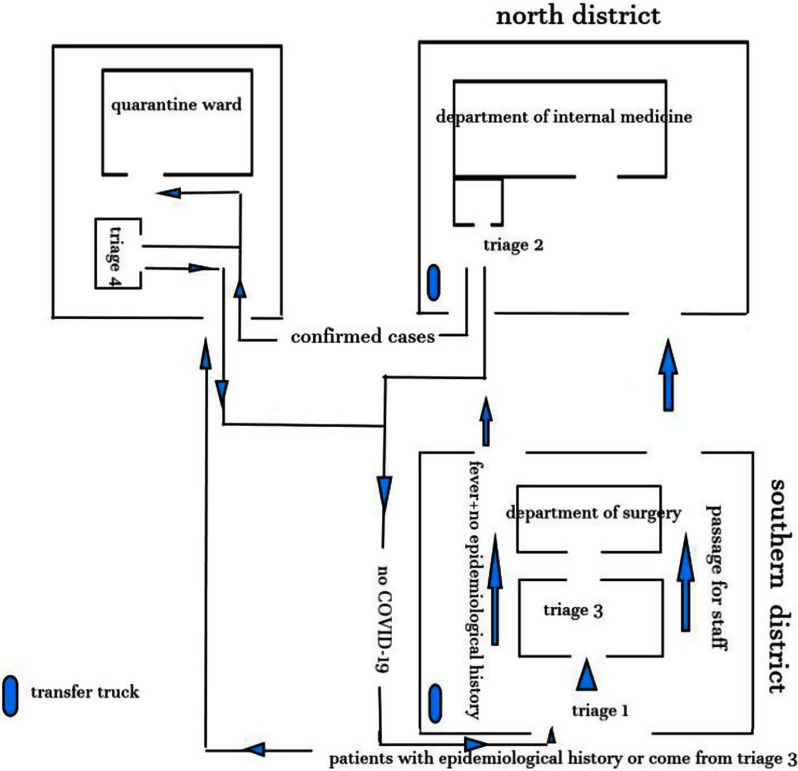
Hospital environmental setting.

All exits of the hospital were closed except for the one-way entrances for the patients and the designated staff path. The triage level 1 prescreening station was placed at the entrance of the main lobby of the outpatient clinic; the triage level 2 prescreening station was placed in the outpatient clinic for general fever; the triage level 3 prescreening was performed by physicians in different departmental clinics; and the triage level 4 prescreening station for COVID-19 was located in the outpatient clinic of the Department of Infectious Disease on Tangfang Street. The hospital area on Tangfang Street was the isolated ward (Fig. 1). Personal protective equipment (PPE) was required for all healthcare workers; it included an N95 respirator, eye protection, gown, mask, and latex gloves, according to the guidelines. The healthcare workers in the isolated ward used a disposable double layer of protective clothing.

### The workflow of prescreening and triage

Before entering the main lobby of the outpatient clinic, each patient at the triage level 1 prescreening station was questioned about his or her epidemiological history, and their body temperature was measured by the medical staff using an infrared thermometer. Patients with fever but no epidemiological history were sent to the outpatient clinic for general fever to receive the initial diagnosis by the medical staff before being diverted to the relevant department. Patients with normal body temperature and no epidemiological history were allowed to enter the outpatient clinic building. Patients with epidemiological history were given medical-surgical masks and were transferred by the medical staff to the COVID-19 outpatient clinic through a fixed route using a special transfer protocol. Before entering the hospital, all medical staff were required to check their body temperature at the first level prescreening triage, and those with normal body temperature could enter through the designated staff path, whereas those with abnormal body temperature were screened according to the patient treatment protocol.

**Fig. 2.**
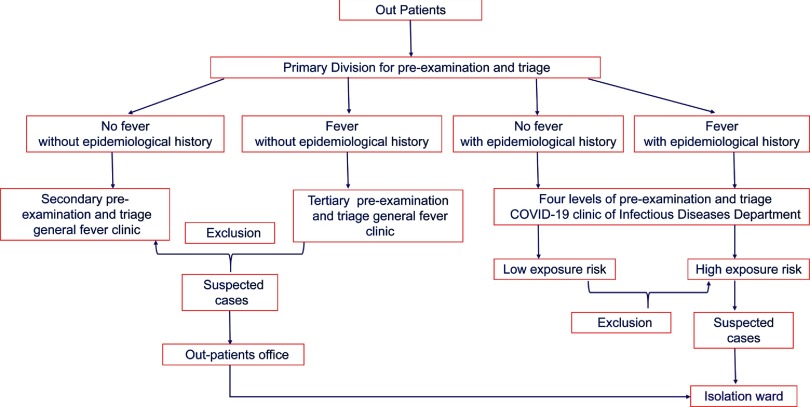
Flowchart of the 4-level triage system.

After nurses measured body temperature and registered the information with the Department of Infectious Diseases, the patients were categorized into 1 of 2 groups: the high exposure-risk group or the low exposure-risk group. They were then guided to their respective waiting areas. Relevant epidemiological history combined with their main complaint, symptoms, and vital signs were carefully prescreened by the physicians of the Department of Infectious Diseases. The blood samples of these patients were collected by the nurses in the department and were transferred to the laboratory further analysis. A chest computed tomography (CT) scan was scheduled by the physicians in the COVID-19 outpatient clinic, who further informed the medical staff in the imaging department to guide these patients through a fixed route in the hospital for imaging and to return to the original waiting area in the COVID-19 outpatient clinic thereafter. The patients who were suspected to have COVID-19 after the prescreening were guided to a quarantine ward for further isolation and monitoring. Patients who were not suspected to have COVID-19 were guided to the main lobby of the outpatient clinic building. All physicians working at different outpatient clinics and physicians in the outpatient clinic for general fever were asked to prescreen and triage the suspected cases according to the established process. Patients who met the criteria for suspected COVID-19 were requested to report to the outpatient office and were transferred by the medical staff to the isolation ward on Tangfang Street for further isolation and monitoring according to the guideline protocol (Fig. 2).

### Screening protocol for suspected cases

The temperature of the patients in the isolation ward was measured twice daily (in the morning and evening), and the changes in lymphocyte level and chest CT were monitored dynamically. A few patients with clinical manifestations of COVID-19 were unable to provide a clear epidemiological history (eg, unclear contact history); they were also admitted to the isolation ward for observation. Nucleic acid testing was carried out in patients during disease progression, and further isolation treatment was carried out in patients with positive nucleic acid tests. If a patient with clinical manifestations of COVID-19 had 2 or more negative nucleic acid tests, the patient’s treatment continued in a single room until his or her body temperature was normal and the chest imaging had returned to normal. After discharge, these patients were advised to continue isolation 14 days at home.

### Statistical analysis

We used SPSS version 23.0 software (IBM, Armonk, NY) for statistical analysis in this study. The Fisher exact test was used to compare the frequency among groups when the theoretical frequency of cells was <5, otherwise, the χ^2^ test was used, with a significance threshold of *α* = 0.05.

## Results

From February 1, 2020, to February 14, 2020, a total of 25,742 triage protocols were completed in the triage level 1 prescreening station of Xi’an Central Hospital; 6,390 triages were performed in general outpatient clinics; and 18,947 staff, inpatients, or visiting staff entered the prescreening and triage process through the outpatient clinic. Also, 246 triage protocols were completed in the triage level 2 station (ie, the outpatient clinic for general fever), and 157 triages were completed in the triage level 4 station (ie, the COVID-19 outpatient clinic). In total, 86 cases were isolated in the quarantine wards, including 18 suspected cases of COVID-19. Among those suspected cases, 3 cases were confirmed by a nucleic acid test. No medical staff were infected, and the rate of missed COVID-19 cases was nil, or 0%.

### Compositions of the COVID-19 outpatient clinic and the outpatient clinic for general fever

Among the 246 patients diagnosed and treated in the outpatient clinic of general fever, fever of unknown origin was the main cause for investigation, followed by upper respiratory tract infections, pneumonia, and other diseases, including urinary tract infections, gingivitis, soft-tissue infection, ankylosing spondylitis, interstitial pulmonary fibrosis, esophageal cancer, and agranulocytosis. Among these patients, 5 patients asked to be screened for COVID-19. Among the 157 patients diagnosed and treated in the COVID-19 outpatient clinic, upper respiratory infection was the main cause of investigation, followed by fever of unknown origin, pneumonia, pulmonary lesion(s), gastroenteritis, coronary heart disease, and cerebral infarction. Also, 14 of these patients asked to be screened for COVID-19 (Table 1).

**Table 1.** Disease Compositions of Outpatient Clinics for General Fever and COVID-19

Disease	Cases in Outpatient Clinic for General Fever, No. (%)	Cases in COVID-19 Outpatient Clinic, No. (%)
Fever of unknown origin	85 (34.6)	37 (23.6)
Upper respiratory tract infection	81 (32.9)	61 (38.9)
Pneumonia	33 (13.4)	31 (19.7)
Request screening for COVID-19	5 (2.0)	14 (8.9)
Bronchitis	10 (4.1)	2 (1.3)
Tonsillitis	10 (4.1)	2 (1.3)
Pharyngitis	2 (0.8)	3 (1.9)
Others	20 (8.1)	7 (4.4)
Total	246 (100)	157 (100)

### Comparison of the screened rate of suspected COVID-19 cases in different departments

Among the 18 suspected cases of COVID-19, 4 patients were referred from the outpatient clinic for general fever, 1 patient was referred from the cardiology outpatient clinic, and 13 patients were referred from the COVID-19 outpatient clinic. All 3 confirmed cases of COVID-19 were referred from the COVID-19 outpatient clinic (Table 2).

**Table 2.** Comparison of the Screened Percent of Suspected Cases of COVID-19 in Different Outpatient Clinics

Clinic	No.	COVID-19 Suspected, No.	% Screened	*P* Value
Outpatient clinic for general fever	246	4	1.63	.00
COVID-19 outpatient clinic	157	13	8.28	
Other outpatient clinics	6,390	1	0.02	

Note: When the theoretical frequency of the cell is <5, it indicates that the χ^2^ test is invalid; therefore, the Fisher exact test method was used instead.

## Discussion

During the severe acute respiratory syndrome (SARS) epidemic in 2003, a large number of medical staff infections prompted medical institutions to take more effective measures to control the epidemic of infectious diseases and hospital infection. Some scholars proposed establishing traffic control, using a triage process for patients, and creating checkpoints for hand washing.^5^ Successful control of the SARS epidemic relied on the integration of several measures, including triage of patients with fever of unknown etiology, mandatory body temperature surveillance, and installation of a fever screening outpatient department.^6^ Infection control measures including hand washing, wearing masks, gloves, wearing protective clothing and shoe covers, and so on, were also recommended by the Centers for Disease Control and Prevention (CDC) in the United States during the SARS outbreak.^7^ These infection control measures were further refined and improved during the Ebola outbreak.^8^ Similarly, the Ministry of Health of China formulated a prescreening and triage system after the SARS epidemic.^9^

Triage is an infection control measure in China. A detailed contact history and occupational history of the patients during the hospital admission process are collected by physicians working in various departments of the medical institutions. Combined with the main complaint, medical history, symptoms, and signs of the patients, prescreening for infectious diseases, those who have been prescreened as patients with infectious diseases or suspected to have infectious diseases should go through the triage process at the Department of Infectious Diseases or another triage point. In addition, necessary disinfection measures should be implemented at the clinics.

When we set up the prescreening and triage process, we fully considered the actual situation at that time. Epidemiological survey results were showing that the major source of viral spread was COVID-19 patients. The early COVID-19 cases in China were all related to the Huanan (Southern China) Seafood Wholesale Market, and the cumulative confirmed cases in Hubei Province and Wuhan accounted for 74.3% and 43.2% of the whole country, respectively, which were the key areas for epidemic prevention and control.^10^ To select patients in urgent need of treatment and to effectively make use of the limited medical resources, medical staff in Wuhan used prescreening and triage strategies based on clinical manifestations.^11^ However, COVID-19 patients in Shaanxi Province mainly migrated from other provinces, where a local epidemic had not yet appeared. Therefore, in nonepidemic areas, the prescreening and triage process should focus on screening infectious sources to prevent the spread of infection in the local area. Therefore, we take the migration and contact history of patients as the key point of screening. According to the environment of our hospital, we set up the triage system shown in Figure 1, and we formulated the prescreening and triage process shown in Figure 2.

Our data show that the total number of screened patients gradually decreased from triage level 1 to triage level 4, while the number of patients suspected of COVID-19 increased. The relevant data from our hospital’s triage showed that the screening rate of suspected COVID-19 in the COVID-19 outpatient clinic was greater than that in the other outpatient clinics. Among these patients, 3 patients with COVID-19 came from the COVID-19 outpatient clinic, suggesting that the triage workflow in our hospital effectively identified the high-risk population and achieved the function of prescreening and triage.

The prescreening and triage process is being adjusted as the epidemic changes. The purpose of this process is to screen out as many potential infectious sources as possible, which may lead to overscreening and overconsumption of medical resources.

Currently, we are still following all staff involved in the prescreening and triage process, but no one has been found to be infected. In a further study, we plan to analyze the protective effect of hand washing, disinfection, and other infection control measures (ICMS) on medical staff in this epidemic.
